# From single fields to river basins: Identification of critical source areas for erosion and phosphorus losses at high resolution

**DOI:** 10.1007/s13280-018-1134-8

**Published:** 2018-12-19

**Authors:** Faruk Djodjic, Hampus Markensten

**Affiliations:** Department of Aquatic Sciences and Assessment, Swedish University of Agricultural Sciences, Lennart Hjälmsv. 9, P.O. Box 7050, 75007 Uppsala, Sweden

**Keywords:** Critical source areas, Distributed modelling, Erosion, High-resolution, Phosphorus

## Abstract

Concentrations of phosphorus (P), the main limiting nutrient in freshwater ecosystems, need to be reduced, but this is difficult due to high spatial and temporal variations and limited resources. Reliable targeting of critical source areas, such as erosion-prone fields and parts of fields, is necessary to improve the cost efficiency of mitigation measures. We used high-resolution (2 m × 2 m) distributed modelling to calculate erosion risk for a large area (202 279 km^2^) covering > 90% of Swedish arable land. Comparison of model results with independent farmers’ observations in a pilot catchment showed high spatial agreement. The modelled worst case scenario produced reasonable quantitative results comparable to measured 90th percentile values of suspended sediment (SS) loads at both field and small catchment scale (*R*^2^ = 0.81, *p* < 0.001). Overall, loads of SS, especially during extreme episodes, strongly governed losses of unreactive P and total P at both field and catchment scale.

## Introduction

Loads of phosphorus (P), the main limiting nutrient in freshwater ecosystems, cause intense algal blooms and impair water quality (Schindler 1974). Identification and limitation of point-source inputs of P to surface waters have been rather successful, whereas nonpoint sources, mainly within agriculture, remain elusive and more difficult to identify, quantify, target and remediate (Sharpley 2016). For instance, in Sweden, emissions of P from wastewater treatment plants have been reduced from 1050 ton in 1987 to 237 ton in 2016, reaching an average treatment efficiency of 96% (Statistics Sweden 2018). Recent estimates of the nutrient loads from Sweden to the Baltic Sea have identified diffuse losses from agriculture as the largest anthropogenic source of P (Ejhed et al. 2016). However, the effects of mitigation programmes focusing on agricultural sources remain difficult to quantify. For most abatement programmes, the key metric of success is the extent to which a practice is implemented, rather than the effectiveness of its implementation in mitigating water quality degradation (Kleinman et al. 2015).

The majority (~ 80%) of diffuse P losses originate from a small proportion of catchment areas (~ 20%), a situation known as the 80:20 rule (Sharpley et al. 2009). These so-called critical source areas (CSAs) coincide with hydrologically active, interconnected areas where overland and/or shallow subsurface flow mobilise and transfer P from terrestrial to aquatic ecosystems. In humid hill-land watersheds, relatively small and well-defined areas typically contribute much of the nonpoint source water, sediment, P and N exported in watershed outflow (Pionke et al. 2000). McClain et al. (2003) coined the term biogeochemical ‘‘hot spots’’ to describe “areas (or patches) that show disproportionately high reaction rates relative to the surrounding area (or matrix).” According to Pionke et al. (2000), it is important to develop concepts, modelling tools and sampling protocols to identify and assess the impact of these CSAs. Identification, quantification and targeting of these CSAs still remain a challenge for the research community and for policy makers. Therefore, despite the extensive body of scientific evidence suggesting that P losses are episodic and spatially variable, current environment protection programmes are not designed to target the most vulnerable parts of the landscape, but applied in a rather general way. Soil erosion is linked to the detachment of soil particles and associated P and provides physical mechanism for mobilising P from soil to water (Haygarth et al. 1999). The infiltration capacities of soils in humid temperate areas are generally high in comparison to rainfall intensities and do not lead to infiltration-excess runoff. However, the downslope flow of water may locally exceed soil storage capacity and result in flow over the surface (Beven and Kirkby 1979). Topography exerts in such cases first-order control on spatial variations in hydrological conditions (Sørensen et al. 2006). The Universal Soil Loss Equation (Wischmeier and Smith 1978) and the Revised Universal Soil Loss Equation (Rennard et al. 1991) are empirical equations for the computation of soil losses in agricultural fields. By considering the influence of flow convergence or divergence (Mitasova et al. 1996) on erosion/deposition processes and replacing slope length (L) and steepness (S) factors with upslope contributing area (Moore and Burch 1986), the modified unit stream power erosion deposition (USPED) model utilises the accuracy of high-resolution DEM to predict the spatial distribution of erosion processes across the watershed. Additionally, the shape of slope has a significant impact on rill patterns, sediment yield and runoff generation (Rieke-Zapp and Nearing 2005).

Recent advances in high-resolution elevation data and new modelling strategies have enabled accurate identification of CSAs at landscape and catchment scale using the modified USPED model with consideration taken to the shape of slope (Djodjic and Villa 2015; Thomas et al. 2016; Djodjic et al. 2018). However, there are two main constraints on these efforts. First, such modelling studies cover rather small areas/catchments, usually those for which more detailed input data are available compared with existing regional or national data. Second, the results of these studies are expressed in relative and qualitative terms, rather than in quantitative terms. In order to be more useful for policy development, modelled risk maps need to be available for a large proportion of a country or a region. Risk maps must also maintain the high resolution needed to take into account spatial variation at field or sub-field scale. With the main objective of improved targeting of CSAs with appropriate countermeasures, especially regarding proper placement of vegetated buffer strips, in March 2017 the Swedish Board of Agriculture commissioned modelling of high-resolution (2 m × 2 m) erosion risk maps for three southern Swedish water districts and the river Dalälven watershed. This area amounts to 202 279 km^2^, which is almost half the total land area of Sweden (407 340 km^2^) and represents 90.4% of Swedish agricultural land. This paper (1) presents the results of the modelling; (2) evaluates the results in terms of their spatial precision regarding identification of observed CSAs for erosion and P losses and their quantitative reliability, by comparing modelled results with measured data from water quality monitoring programmes; and (3) examines the importance of erosion and suspended sediment (SS) loads for losses of P in general, and unreactive particulate P (UP) in particular.

## Materials and methods

**Fig. 1:**
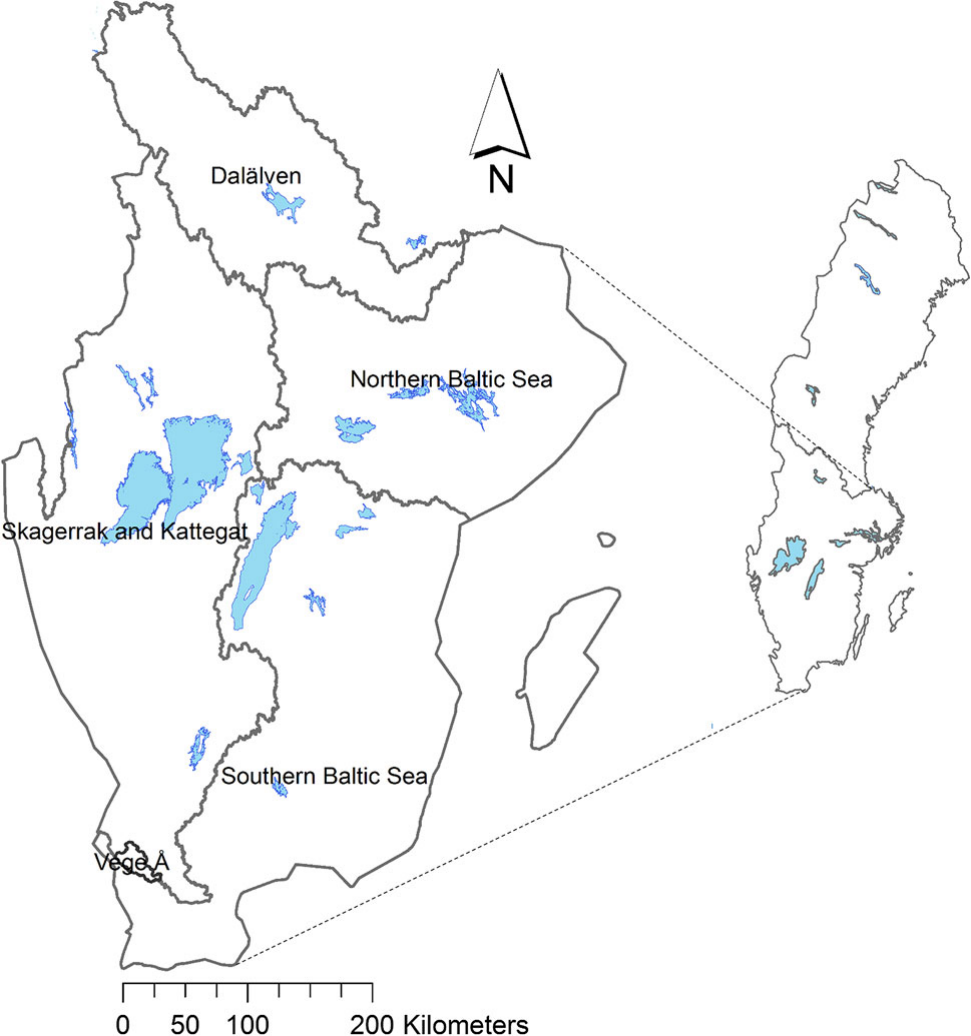
Map of Sweden showing modelled areas (the three southern Swedish water districts, Dalälven) and the Vege å catchment

The modelled area, covering three southern water districts (Southern Baltic Sea, Northern Baltic Sea and Skagerack and Kattegat) and Dalälvens catchment is shown in Fig. 1. The project was carried out in two phases. In the first phase, the distributed high-resolution modelling was performed and erosion risk maps were created for Vege å catchment (488 km^2^) in south-west Sweden (Fig. 1). The erosion risk maps were then independently evaluated by the Swedish Board of Agriculture through visits to farmers in the Vege å catchment and comparisons with farmers’ own observations of erosion and overland flow. Success in this phase, specified as good agreement with independent observations of erosion and overland flow by farmers in the area, was a precondition for continuation of the project and successive modelling of the rest of the area.

The basis for the modelling work was a digital elevation model (DEM) in raster format. A 2-m grid based on LiDAR data was used, with a density of 0.5–1 point m^−2^ and accuracy usually better than 0.1 m (Lantmäteriet 2014). The modified USPED model (Mitasova et al. 2001) was implemented within a frame of PCRaster software for environmental modelling (Schmitz et al. 2009). In brief, USPED is a simple model which predicts the spatial distribution of erosion and deposition patterns based on the change in overland flow depth and the local geometry of terrain, including both profile and tangential curvatures. The slope length factor (LS) of the RUSLE equation is replaced with upslope contributing area in the modified model and the LS factor is calculated as follows:

1 $$ LS = \left( {\frac{A}{22.13}} \right)^{1.6 } \cdot(\sin b)^{1 + p} $$

where *A* is the upslope contributing area (m^2^) and *b* is the slope angle (degrees). An exponent value of 1.6 was used, as recommended by Mitasova et al. (2001), while the value of exponent *p* depends on soil texture and describes soil permeability (Table 1). Thereafter, slope profile (*ProfCurv*) and tangential curvature (*TanCurv*) calculated from DEM were used to account for the effect of slope form on erosion and deposition patterns. Uniform, nose and convex linear slopes yield more sediment than concave linear and head slopes, where sediment is deposited on toe slopes (Rieke-Zapp and Nearing 2005). To account for these patterns, erosion/deposition (ED) was calculated as follows:

2 $$ ED = R*LS*K*C*\left( {1\, + \, - 1*ProfCurv} \right) * \left( {1 + {-}1 * TanCurv} \right) * 4 $$

where *R* is erosivity factor (here average water discharge, mm), *K* is soil erodibility factor (t ha^−1^), *C* is vegetation cover factor and 4 is a scaling factor (equal to map resolution, 2 × 2 = 4 m^2^). In the modified USPED, *R*, *K* and *C* were applied as described below. According to Eq. 2, convex parts of the landscape (negative profile curvature) are assigned positive values, indicating net erosion, while concave parts of the landscape (positive profile curvature values) are assigned negative values, indicating net deposition. The same approach applies for the tangential curvature: according to Eq. 2, positive values of tangential curvature (laterally convex, resulting in diversion of flow) are assigned negative values, indicating net deposition, whereas negative values of tangential curvature (laterally concave, resulting in concentration of flow) are assigned positive values, indicating net erosion. Consequently, each grid cell is assigned a positive net erosion value or negative net deposition value. Finally, in the last step, the accuflux operation in PCRaster is used to calculate for each cell the accumulated amount of material that flows out of the cell into its neighbouring downstream cell. This accumulated value is the amount of material in the cell itself, plus the amount of material in cells upstream of the cell. The local drain direction network, with flow directions from each cell to its steepest downslope neighbour, based on high-resolution DEM is used to accumulate eroded material along the flow paths.

**Table 1:** Values of soil erodibility factor (K) and exponent *p* for different soil categories. *New values introduced to improve modelling results

Soil	Land use	Value of *K*	Value of exponent *p*
Sand	Arable land	0.04	0.75
Loamy sand	Arable land	0.09	0.65
Sandy loam	Arable land	0.1	0.6
Sandy clay loam	Arable land	0.15	0.55
Loam	Arable land	0.5	0.35
Silt loam	Arable land	0.82	0.1
Silt	Arable land	0.9	0.1
Sandy clay	Arable land	0.35	0.45
Clay loam	Arable land	0.67	0.2
Silty clay loam	Arable land	0.95 (0.75*)	0.01 (0.15*)
Silty clay	Arable land	0.82	0.05
Clay	Arable land	0.67	0.15
Organic soil	Other	0.01	0.75
Clay	Other	0.57	0.25
Gravel	Other	0.02	0.75
Cobbles to boulders	Other	0.02	0.2
Fluvio-glacial sediment, sand-block	Other	0.02	0.75
Clay till	Other	0.3	0.3
Till (moraine)	Other	0.1	0.45
Thin soil layer	Other	0.1	0.1
Rock	Other	0.01	0.1
Artificial fill	Other	0.02	0.75
Other	Other	0.02	0.75
Water	Other	0.001	0.99

**Fig. 2:**
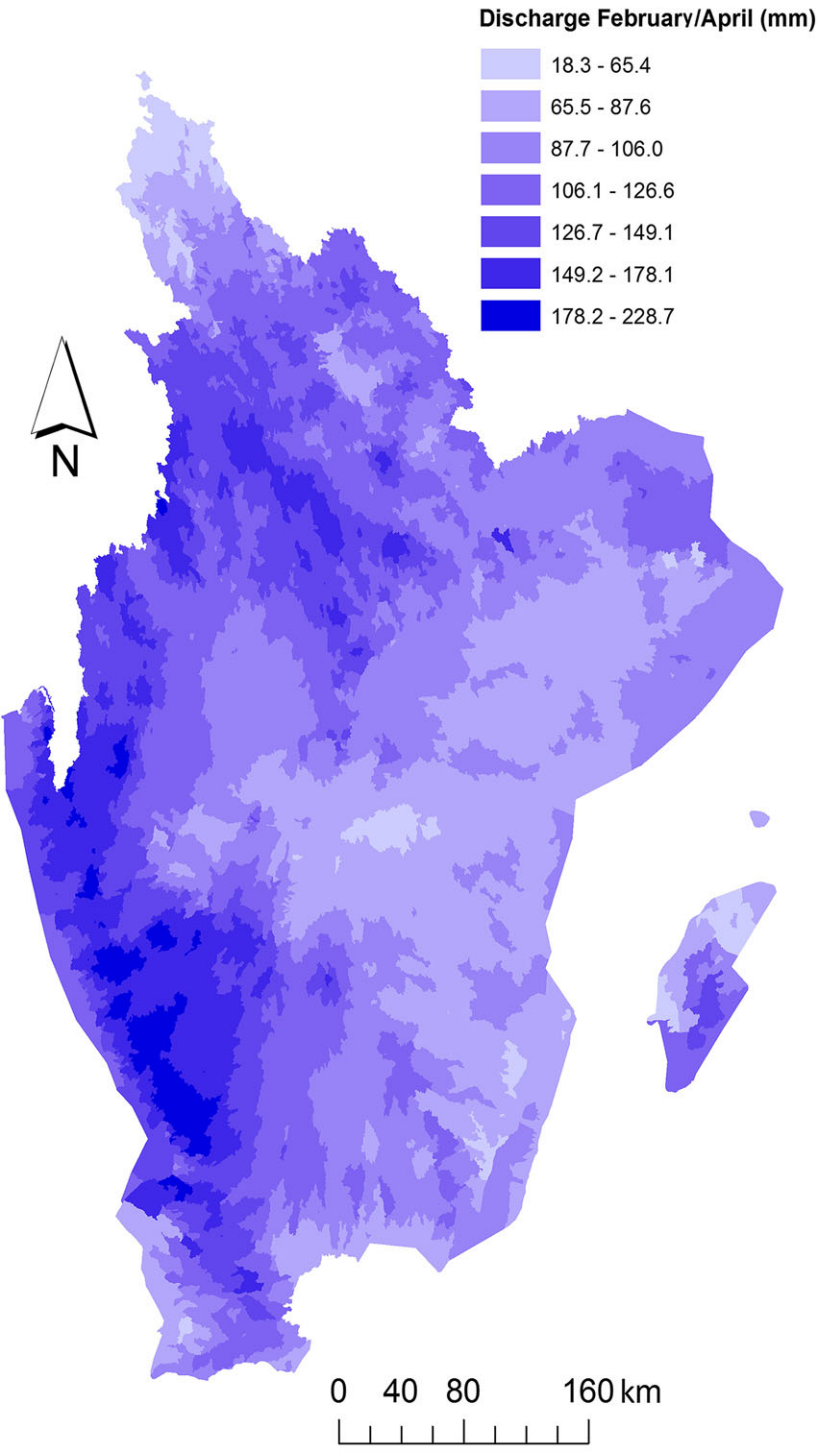
Sum of long-term (1994–2013) monthly average water discharge February to April in 7587 sub-catchments, based on data from the Sixth Pollution Load Compilation project (Ejhed et al. 2016)

**Fig. 3:**
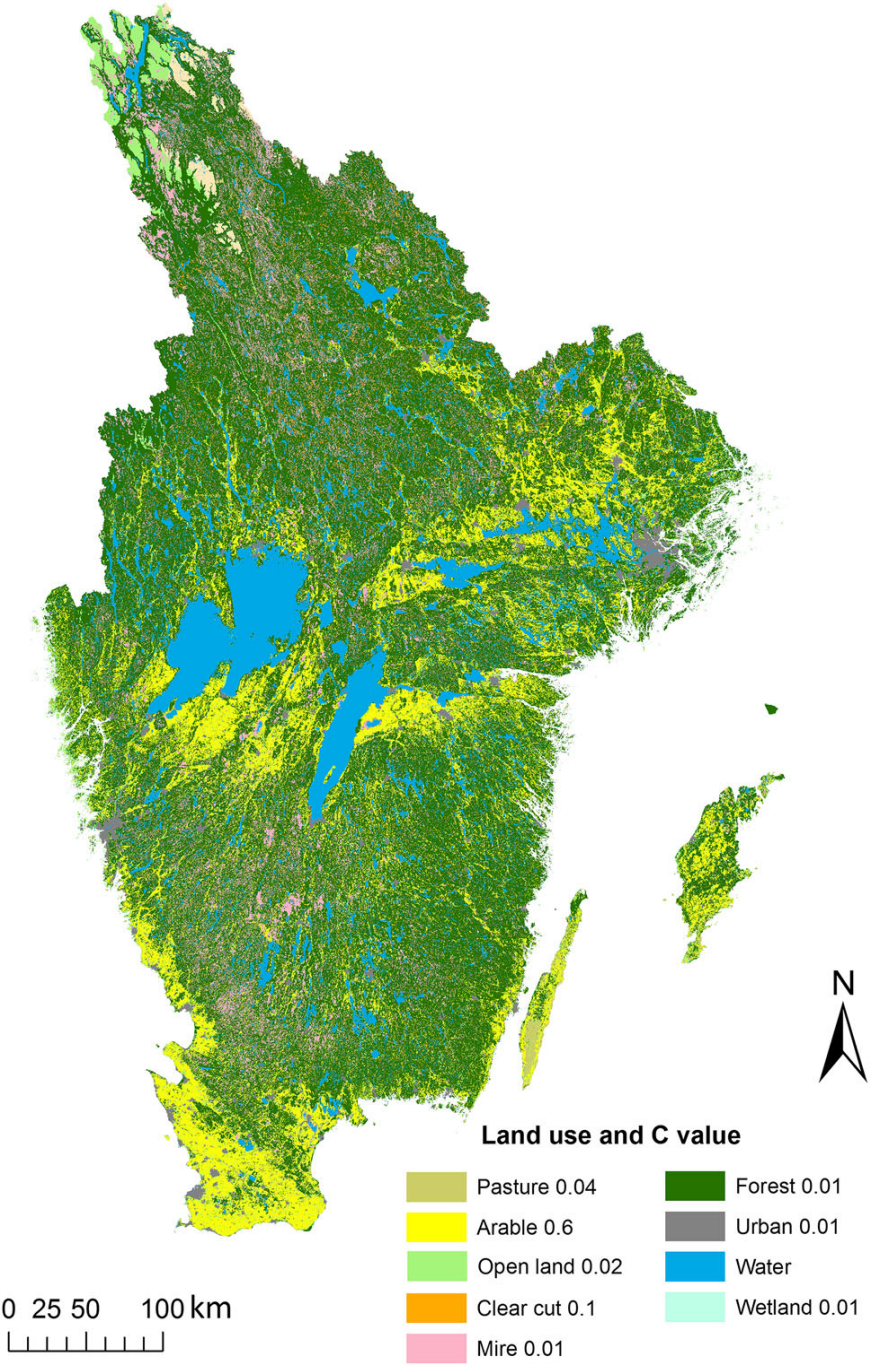
Land use map and corresponding values of crop factor (*C*) for each land use category. The map is based on the land use map developed in the Sixth Pollution Load Compilation project (Ejhed et al. 2016), modified to differentiate between pasture and arable land

The high-resolution distributed modelling of risk maps for erosion was performed as a “worst case scenario,” which governed the selection of input data and parameters. First, erosion in Sweden usually occurs during winter-spring season, with snowmelt in particular being a critical factor for erosion (Alström and Bergman 1990; Brandt 1990). Therefore, the sum of long-term (1994–2013) monthly average water discharge for February to April in 7587 subcatchments (Fig. 2) was used as the monthly erosivity factor (R). The specific runoff (mm or l m^−2^) was assumed for the whole catchment, i.e., no differences in specific runoff were assumed between land use categories. As described earlier, the rainfall intensity in Sweden is rather low in comparison with the soil infiltration capacities and the erosion occurs usually due to the soil water saturation. Therefore, use of water discharge has been shown to give reasonable estimates of erosion (Djodjic and Villa 2015), as high flow episodes result in high losses of both SS and TP. Second, the effects of vegetation cover factor (*C*) were determined based on a national map of land use distribution (Fig. 3). The land use map from the Sixth Pollution Load Compilation project (PLC6) was modified using data from the Swedish Board of Agriculture on agricultural blocks under pasture, in order to differentiate between pasture and arable land. In the PLC6 map, these two land uses are one category. The base map for the PLC6 map is GSD-Road map 1:100 000 from 2013 (Widen-Nilsson et al. 2016) improved to better account for clear cuts, agricultural land and urban areas (1:10 000). Since the situation modelled was designed as the “worst case scenario,” all arable land was assigned the same and high C factor value (0.6), in order to avoid annual differences due to crop rotations. For instance, in Sweden, the largest crop is ley (grasslands) accounting for almost half (48%) of the arable area (Johnsson et al. 2016) followed by spring barley 16% *(Hordeum vulgare* L*.)*, winter wheat 9% *(Triticum aestivum)* and oats 9% *(Avena sativa)*. Grassland crop offers a good protection from erosion but these leys are not permanent grasslands and these fields are included in the crop rotation and regularly ploughed. Therefore, the actual crop distribution for a single year would result in lower erosion rates on fields where leys are grown, but which might be ploughed up already next year.

**Fig. 4:**
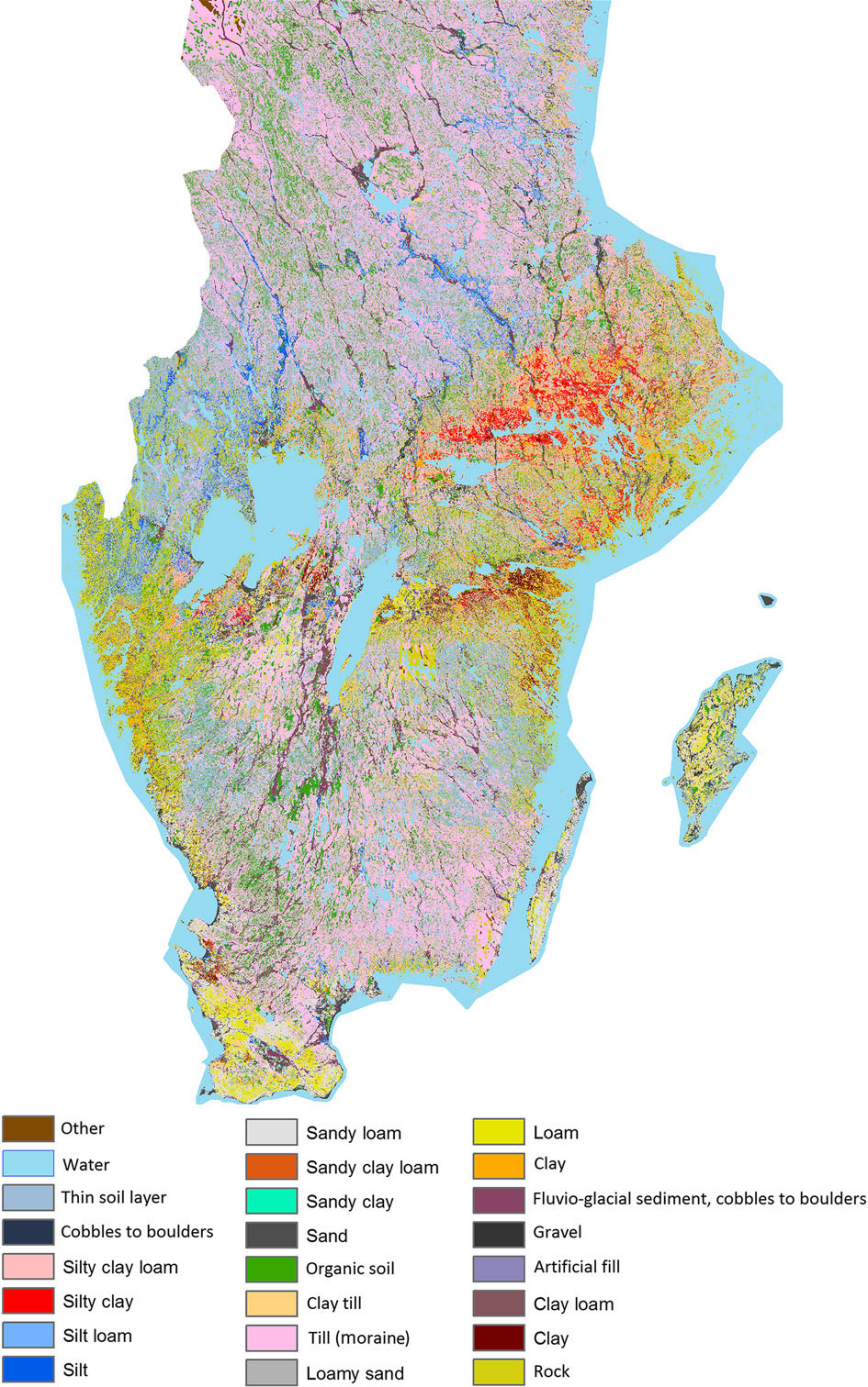
Soil texture and other materials distribution map used for erosion modelling. The map is based on the Digital Arable Soil Map of Sweden (Söderström and Piikki 2016) for agricultural land and soil maps from the Geological Survey of Sweden for non-agricultural areas

The values of soil erodibility factor (K) and values of exponent *p* in Eq. 1 were based on the new soil map of textural classes of Swedish agricultural soils (Söderström and Piikki 2016), in combination with soil maps from the Geological Survey of Sweden for non-agricultural areas (Fig. 4). The Digital Arable Soil Map of Sweden, DSMS (Söderström and Piikki 2016) is a 50 m × 50 m raster, whereas the map from the Geological Survey of Sweden for non-agricultural areas is a combination of the best available data with a spatial resolution ranging from 1:50 000 to 1:250 000.

All above-mentioned maps were transformed to 2 m × 2 m raster layers to make them compatible with the high-resolution DEM layer. The modelled area was divided into 784 catchments (ranging from < 1 to 709 km^2^), for which the model was then run in succession.

Two main outputs were obtained from the model. First, net erosion or deposition (kg/ha) was calculated for each raster cell (2 m × 2 m) according to Eq. 2. Second, eroded material was accumulated along the flow accumulation lines based on the flow direction maps. Hence, overland flow and erosion lines were calculated for all raster cells along the flow accumulation lines with upstream areas exceeding 5 hectares. The 5 ha threshold was chosen as a compromise between a need to highlight main erosion trajectories and the need to keep the data volumes at reasonable and manageable levels. After quantitative modelling of erosion, the results were processed to create the erosion risk classes requested by the Swedish Board of Agriculture.

The modelling results were evaluated in two different ways. First, the spatial distribution of erosion was compared against farmers’ observations of erosion and overland flow traces. This evaluation was performed by the Swedish Board of Agriculture through individual visits and interviews with six farmers in the pilot catchment of Vege å. Prior to the visits, farmers received high-resolution aerial photographs of their fields and were asked to draw and document the areas where they have experienced problems with overland flow, erosion and ponding waters. Observations drawn on maps by farmers were then digitised for comparison with the modelled values. Here, we illustrate the agreement between modelled and observed erosion areas using available digital maps for the farms of two of these farmers with adjacent fields.

Second, the accumulation of the eroded material along the flow lines enabled model evaluation and comparisons with measured data of SS loads recorded in Swedish water quality monitoring programmes (Table 2).

**Table 2:** Fields and catchments included in water quality monitoring programmes used for the quantitative evaluation of modelling results, their official code and their area, dominant soil textural class for arable land and percentage of arable land

ID	Type	Area (ha)	Dominant arable soil textural class	% arable land
21E	Field	4.5	Sandy loam	100
1D	Field	6.3	Silty clay loam	100
3M	Field	8.7	Sandy loam	100
20E	Field	10.3	Clay	100
6E	Field	10.8	Loam	100
5O	Field	12.9	Silt loam	100
12N	Field	14.2	Sandy loam	100
4O	Field	19.3	Silty clay loam	100
11M	Field	22.3	Silty clay loam	100
7E	Field	27.0	Silty clay	100
2M	Field	36.4	Loam	100
F26	Catchment	182	Sandy loam	70
I28	Catchment	479	Loam	84
U8	Catchment	574	Silty clay	56
E24	Catchment	626	Clay	66
N33	Catchment	664	Loam	87
M39	Catchment	680	Loam	83
H29	Catchment	753	Loam	65
E23	Catchment	755	Clay loam	54
O14	Catchment	766	Silty clay loam	71
K31	Catchment	769	Loam	25
M36	Catchment	789	Sandy loam	86
M42	Catchment	826	Loam	93
K32	Catchment	947	Loamy sand	66
O17	Catchment	967	Sandy loam	55
N34	Catchment	1393	Sandy loam	85
E21	Catchment	1632	Loam	89
T9	Catchment	2575	Silty clay	45
C6	Catchment	3298	Silty clay	39
S13	Catchment	3522	Silt loam	59

Since the focus of the whole project was on calculating erosion from agricultural land, in model evaluation we used results from two water quality monitoring programmes focusing on the influence of agricultural practices on water quality: (a) Observation fields on arable land (Djodjic and Bergström 2005; Linefur et al. 2017) and (b) the agricultural monitoring programme (Kyllmar et al. 2014). In short, losses of nutrients and SS have been monitored at field scale (4–36 ha) since the early 1970s and at small agricultural catchment scale (2–35 km^2^) since the early 1990s. Data on transported monthly loads of nutrients and SS for both fields and catchments in the period 2000–2016 were downloaded from a database held at the Department of Soil and Environment, Swedish University of Agricultural Sciences (SLU 2018). Total P was analysed on unfiltered samples after digestion in an acid persulfate solution, while dissolved reactive P (DRP) was measured after filtration with a 0.2-µm pore diameter filter (Scheleicher and Schüll GmbH, Dassel, Germany). Suspended solids were determined by filtration using the same filters (i.e., 0.2 µm), as the increase in filter weight. Total P, DRP, and SS analyses were performed according to European standard methods (European Committee for Standardization 1996). Unreactive P (UP) was calculated as the difference between TP and DRP.

This dataset was rearranged and analysed to estimate and evaluate the relevance of modelling results for losses of SS and losses of TP and UP. The 90th percentile of SS loads was first calculated for each field and catchment, based on the assumption that these values would represent the worst case scenario modelled with the modified USPED model. To highlight the relevance of SS loads for P losses, the relationship between average monthly SS loads and loads of TP and PP was then explored for 11 fields and 19 small agricultural catchments. Finally, to illustrate the importance of high intensity episodes for total SS and P transport during the study period, all transported amounts exceeding the 90th percentile value were added together and expressed as percentage of total load during the whole period.

A list of the fields and catchments included in this analysis, together with calculated 90th percentile, are given in Table 3. The goodness of fit was tested with linear regression line and a comparison with the 1:1 line.

**Table 3:** Average annual losses of suspended sediment (SS), monthly 90th percentile of SS and average monthly loads of suspended sediment (SS), total P (TP) and unreactive P (UP) in 11 fields and 19 small catchments

ID	Type	Average annual load	Monthly 90th percentile	Average monthly load		
		SS (tkm^−2^)	SS (tkm^−2^)	SS (tkm^−2^)	TP (kgkm^−2^)	UP (kgkm^−2^)
21E	Field	0.57	0.13	0.04	0.25	0.13
1D	Field	39.71	11.8	4.09	7.87	5.79
3M	Field	1.04	0.18	0.09	13.65	1.09
20E	Field	17.02	3.99	1.47	2.07	1.06
6E	Field	1.53	0.16	0.18	0.48	0.29
5O	Field	5.37	1.61	0.65	1.50	0.97
12N	Field	2.57	0.53	0.19	0.82	0.53
4O	Field	11.15	4.11	1.25	2.88	2.17
11M	Field	51.95	20.34	7.87	8.13	7.37
7E	Field	26.97	10.76	3.74	5.85	3.64
2M	Field	4.72	1.47	0.47	1.52	0.89
F26	Catchment	8.34	1.64	0.88	4.72	2.45
I28	Catchment	1.93	0.50	0.21	1.94	0.48
U8	Catchment	21.88	7.60	2.77	5.92	4.14
E24	Catchment	34.26	7.93	2.96	5.03	2.94
N33	Catchment	6.96	2.69	0.97	4.20	2.26
M39	Catchment	3.69	1.34	0.41	3.21	1.20
H29	Catchment	6.08	0.23	0.10	1.03	0.41
E23	Catchment	16.58	4.97	1.88	4.15	2.13
O14	Catchment	14.69	3.98	1.42	4.44	2.56
K31	Catchment	2.86	0.63	0.22	1.36	0.66
M36	Catchment	19.01	6.28	1.94	4.42	2.71
M42	Catchment	4.58	1.04	0.42	3.03	1.11
K32	Catchment	1.44	0.23	0.10	2.31	1.56
O17	Catchment	2.42	0.41	0.21	1.68	0.73
N34	Catchment	10.88	2.27	0.96	3.08	2.14
E21	Catchment	2.18	0.43	0.20	0.85	0.33
T9	Catchment	31.56	8.45	3.26	7.48	4.98
C6	Catchment	29.02	8.51	2.65	3.82	2.61
S13	Catchment	10.97	2.45	0.86	2.86	1.50

The modified USPED model was not calibrated in the real sense of the word. Instead, the parameter values shown in Table 1 were selected based on previous studies, experience/expert judgement and the literature (Djodjic and Villa 2015; Djodjic et al. 2018). However, a simple procedure was performed to evaluate and illustrate the sensitivity of the model to parameter values of R, LS, K and C. A representative range of all above-mentioned parameters was chosen and erosion values were calculated based on Eq. 2 for all combinations of parameter values from this matrix.

## Results

**Fig. 5:**
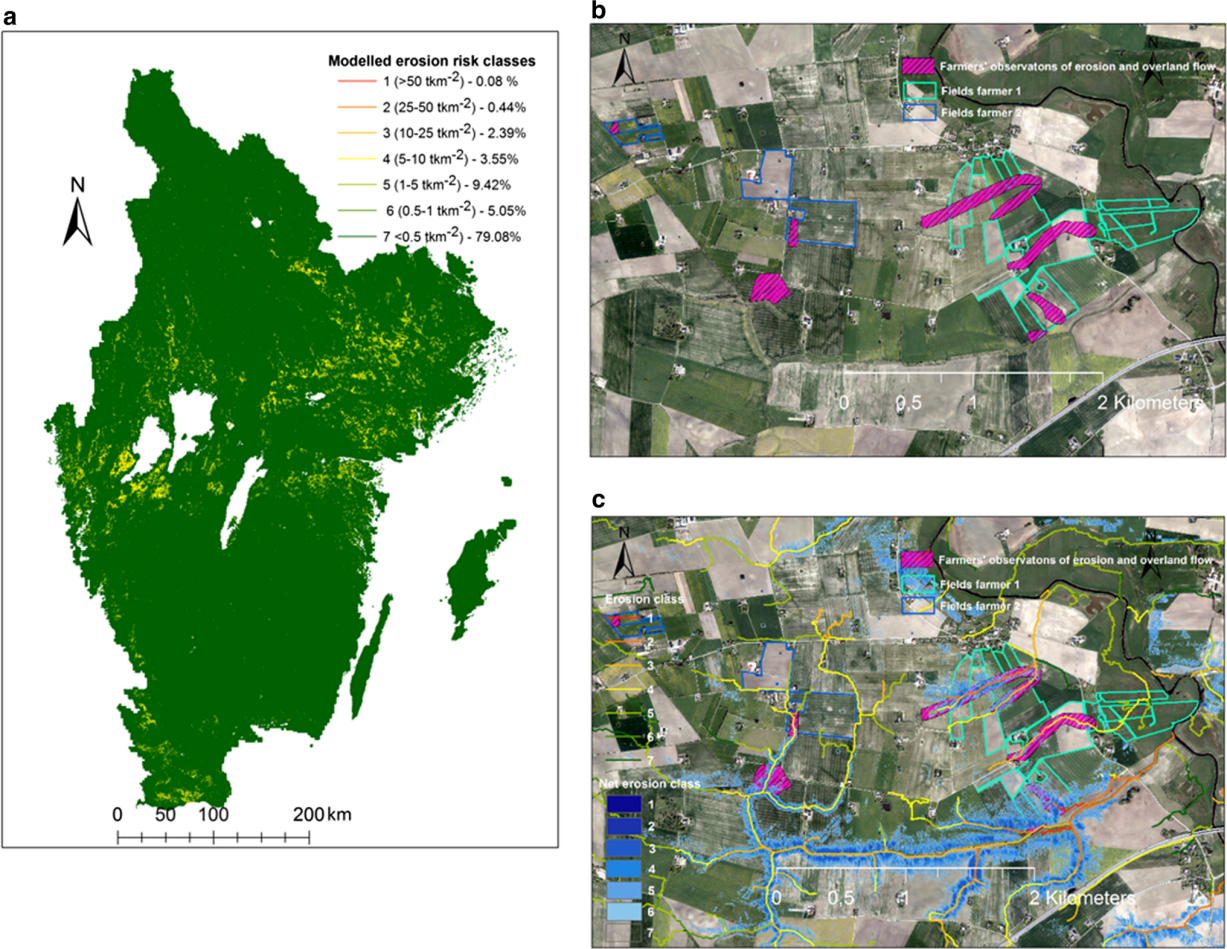
**a** Modelled monthly, worst case scenario erosion risk classes for the whole modelled area with a percentage of modelled area in each erosion risk class, **b** observed risk areas for overland flow and erosion identified by farmers and **c** high-resolution modelling results (net erosion and erosion accumulation lines) for the same area as in (**b**). Both net erosion and erosion accumulation lines in (**c**) have the same unit (t km^−2^) and class division as in (**a**)

The total modelled area (202 279 km^2^) and the high-resolution (4 m^2^) meant that more than 50 × 10^9^ cells were processed to create erosion risk maps. Figure 5a shows the final results for the whole modelled area. Due to generally low erosion in Sweden, the erosion classes in Fig. 5a were defined to make possible illustration of differences at the lower end of erosion range. For instance, the class divisions of monthly erosion presented here are approximately two orders of magnitude lower than annual erosion classes presented by Panagos et al. (2015). In spite of that, more than 79% of the total modelled area was included in the lowest erosion class (< 0.5 tkm^−2^). According to the modelling results, only 2.91% of the total area was included in the three highest erosion classes (> 10 tkm^−2^), while the remaining 18% was in erosion classes 4–6.

The real strength of the modelling results is the possibility to zoom in on any given field/part of field and obtain high-resolution data, as shown in Fig. 5b and c. The farmers interviewed by the Swedish Board of Agriculture were able to delineate known parts of their fields where overland flow and erosion usually take place (Fig. 5b). The shape and size of these identified high-risk areas varied, with the smallest being just ~ 0.5 ha and the largest being 8.8 ha and stretching over several fields. In total, 24.3 ha (20%) out of a total field area of 121.8 ha belonging to the two interviewees selected as examples were identified by those farmers as areas prone to erosion and overland flow. In addition, one area (5.2 ha large polygon in the south-west) outside their own fields was identified as vulnerable. Note that the lowest net erosion class (class 7) in Fig. 5c is transparent. There was good spatial agreement between the farmers’ observed erosion areas and the modelled net erosion and erosion accumulation lines (Fig. 5c). The erosion accumulation lines run straight through the observed risk areas, and also indicate how different observed erosion areas are connected in the landscape. However, the highest erosion according to the model takes place outside the farmers’ fields, in the southern part of the area in Fig. 5c, around a small stream running through the region. Coincidentally or not, that small stream is called Clay Creek (*Lerbäcken* in Swedish), indicating frequent occurrence of turbid, muddy water, probably a consequence of the high erosion in its catchment.

**Fig. 6:**
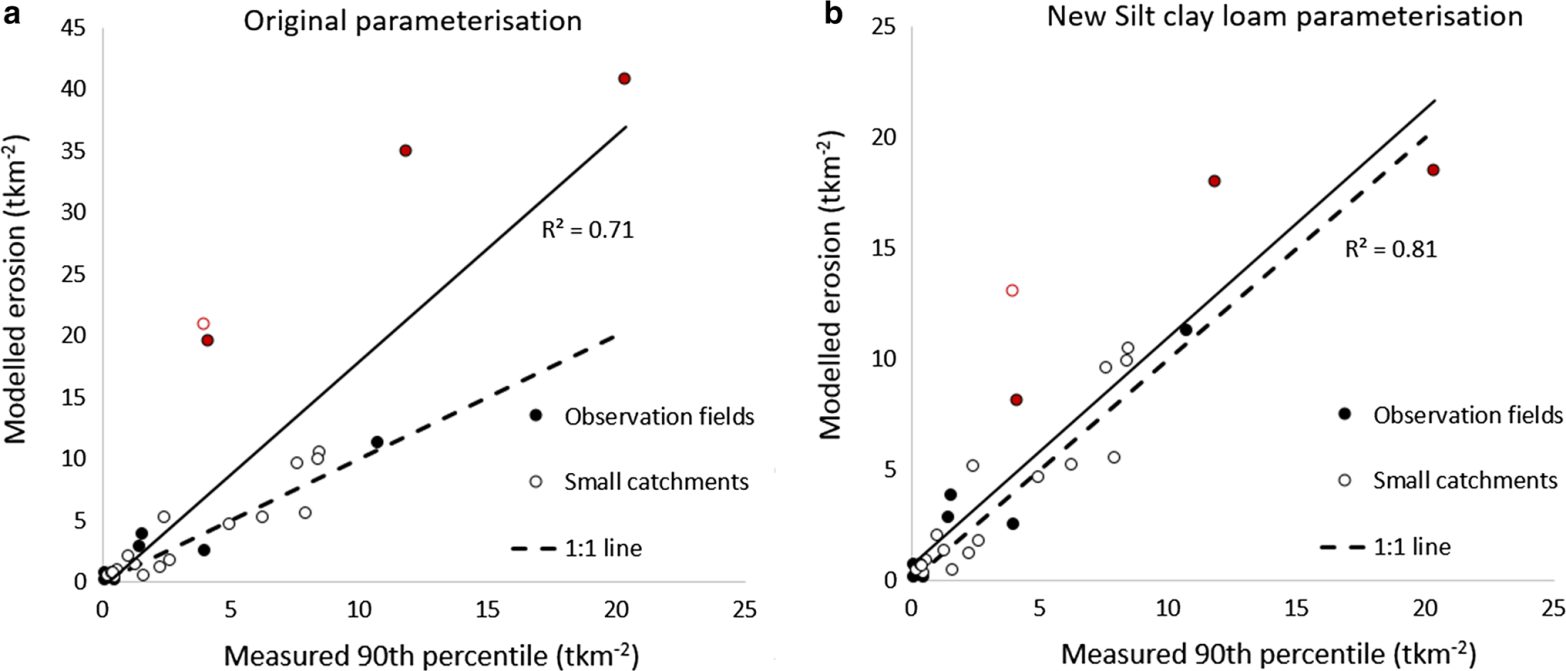
Linear regression relationship between 90th percentile of measured SS monthly loads for 11 fields and 19 small catchments and modelled erosion with **a** original parameter values and **b** adjusted parameter values (*K*, *p*) for silty clay loam soil

**Fig. 7:**
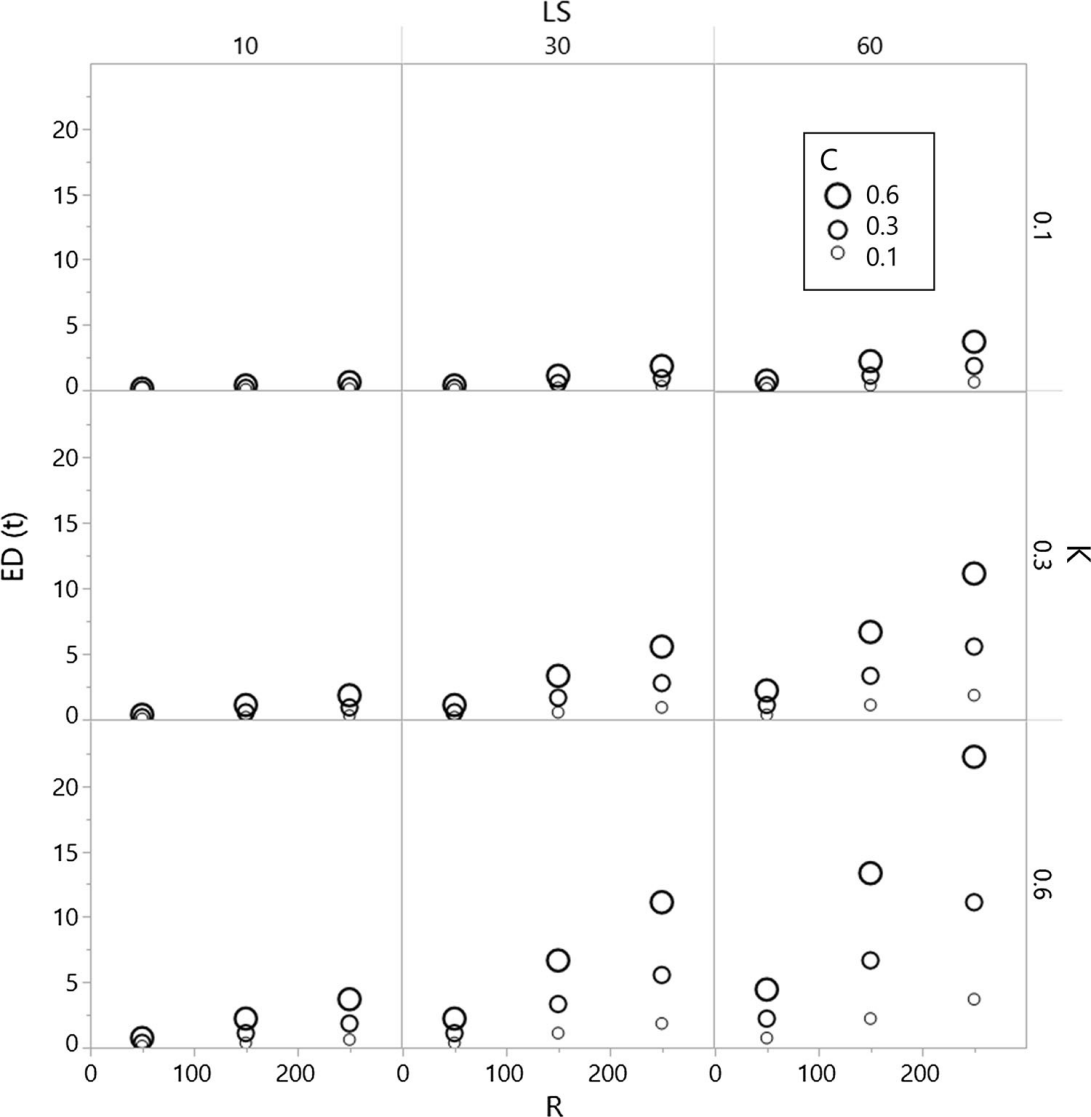
The dependence of calculated erosion values (primary *y*-axis) on four important factors: the erosivity factor (R, primary *x*-axis), the slope length factor (LS, secondary *x*-axis), the soil erodibility factor (K, secondary *y*-axis) and vegetation cover factor (*C*, size of the marker)

Quantitative comparison of modelled results with the 90th percentile of measured monthly loads of SS from the 11 observation fields and 19 small agricultural catchments listed in Table 3 revealed a strong positive linear relationship between measured and modelled values (*R*^2^ = 0.71, *p* < 0.001) (Fig. 6a). Coincidentally, the 90th percentile values assumed as approximations of the modelled worst case scenario were close to the 1:1 line (dotted line), but with three fields and one catchment (red circles in Fig. 6) deviating from this pattern by having considerably higher modelled values compared with the measured 90th percentile. The common denominator for these three observation fields (1D, 4O and 11M) and one small catchment (O14) is silty clay loam as the dominant soil textural class (Table 2). Therefore, a simple test was performed to see whether changes in parameter values improved model performance for some of the fields/catchment deviating most from the general pattern. This was done by changing parameter values *K* and *p* for the silty clay loam textural class according to Table 1, using the new values in brackets, and then repeating the modelling for the sub-catchments in which these fields/small catchments were located. Villa (2014) showed that the Swedish medium fine textured soils had the highest mean value for both soil dispersion and calculated K value, but it was difficult to assess the differences between individual soil textural classes within this broader class. Therefore, the high values for the silty clay loam were decreased to be closer to other surrounding textural classes such as clay loam and silty clay. Decreasing rather extreme parameter values of K and *p* for silty clay loam (Table 1, new *K* and *p* values in brackets) resulted in further improvement of the linear regression relationship (*R*^2^ = 0.81, *p* < 0.001) (Fig. 6b). This raises the question of how sensitive the modelled erosion values are to a certain parameter values. In Fig. 7, the effect of four main factors (erosivity factor (*R*), the slope length factor (LS), the soil erodibility factor (*K*) and vegetation cover factor (*C*) on calculated erosion values are presented. It is clear that fields in the upper left part of Fig. 7, with low LS factor and low *K* factor (such as sandy soils, Table 1), will have low erosion values, irrespectively of erosivity (*R*) value and crop cover factor. As we move towards the lower right part of Fig. 7, the increasing LS and *K* values lead to increasing erosion values. At the same time, the importance of *C* factor also increases, where low *C* factor (smallest markers) reduces erosion substantially.

A very strong positive linear relationship between average monthly loads of SS and UP (Table 3) was found for all 19 small agricultural catchments (*R*^2^ = 0.81, *p* < 0.001) and 11 observation fields (*R*^2^ = 0.93, *p* < 0.001) included in this study. A strong positive linear relationship was also found between SS and TP for the 19 small agricultural catchments (*R*^2^ = 0.74, *p* < 0.001) and 10 of the 11 observation fields (*R*^2^ = 0.87, *p* < 0.001) (the sandy field 3 M, heavily dominated by losses of dissolved P (DP), was excluded).

Analyses of the monthly transport values exceeding the 90th percentile of SS loads (Table 4) showed that these months, which represented 10% of the total number of months in the study period (2000–2016), delivered around 30% of total water discharge (on average 32% for observation fields and 29% for small catchments, but were responsible for more than half the total load of SS (on average 62% for observation fields and 52% for small catchments). These months with the highest SS loads also carried a high proportion of total loads of PP (on average 53% for observation fields and 41% for small catchments) and TP (on average 49% for observation fields and 37% for small catchments).

**Table 4:** Water discharge (Q), suspended sediment (SS), total phosphorus (TP) and unreactive phosphorus (UP) during months exceeding the 90th percentile of SS monthly loads, expressed as percentage of total volume (Q) or load (SS,TP,UP) in 11 fields and 19 small catchments during the period 2000–2016

ID	Type	Q (%)	SS (%)	TP (%)	UP (%)
21E	Field	35	60	51	58
1D	Field	33	55	43	49
3M	Field	24	45	24	24
20E	Field	41	82	74	76
6E	Field	38	89	74	80
5O	Field	29	69	55	62
12N	Field	26	42	36	34
4O	Field	28	49	37	39
11M	Field	35	57	51	54
7E	Field	30	68	45	53
2M	Field	34	69	52	58
Field average		32	62	49	53
F26	Catchment	21	52	28	33
I28	Catchment	37	25	44	48
U8	Catchment	27	70	38	41
E24	Catchment	28	58	40	47
N33	Catchment	23	54	29	37
M39	Catchment	29	37	40	49
H29	Catchment	41	40	59	48
E23	Catchment	27	51	32	37
O14	Catchment	24	58	32	39
K31	Catchment	31	49	44	43
M36	Catchment	29	61	34	37
M42	Catchment	31	37	35	41
K32	Catchment	32	67	38	42
O17	Catchment	28	43	31	34
N34	Catchment	23	69	31	32
E21	Catchment	31	55	39	44
T9	Catchment	31	55	39	44
C6	Catchment	32	54	45	49
S13	Catchment	24	52	34	37
Catchment average		29	52	38	41

## Discussion

Studies of erosion in Sweden have been limited to date and no general quantification has been carried out (Ulén 2006). The erosion values in the southern half of the Sweden estimated in this modelling study were generally low (Fig. 5a), which is in line with earlier estimates made at coarser scale and resolution (Panagos et al. 2015). The lower and upper limit of tolerable soil erosion for conditions prevailing in Europe is approximately 0.3 and 1.4 ton ha^−1^ year^−1^, respectively (Verheijen et al. 2009). This means that mean annual transport of SS from some of the fields and catchments included in this study (e.g., 1D, 11M, E24 and T9) exceeds the lower limit of tolerable soil erosion (Table 3). These fields and catchments are dominated by silty clay and silty clay loam soil textures (Table 2), and have high *K* values (Table 1) which creates preconditions for high erosion (Fig. 7). The modelled worst case scenario monthly values of transported SS and the 90th percentile of measured values were below 20 tkm^−2^ (i.e. 0.2 tha^−1^ month^−1^) (Fig. 6b). However, even though the soil loss itself might be tolerable, the transported soil particles act as an important carrier of P from arable land (Ulén 2006). As shown by the highly significant positive linear regression between loads of SS and different P forms (TP and PP), transport of SS governs transport of PP and, more often than not, transport of TP. There was one exception to this pattern in the dataset used here, which was related to high losses of DP from sandy, non-erosive soil (e.g., field 3M) with low P sorption capacity (Djodjic and Bergström 2005). Therefore, identification and targeting of erosion and overland flow losses in P-balanced systems such as Swedish agriculture (Djodjic et al. 2005; Bergström et al. 2015) might be one of the rare low-hanging fruits still available to mitigation efforts. The problem to date has been identification of erosion-prone areas at field and sub-field scale. We found high spatial agreement between modelled erosion-prone areas and observed occurrences of erosion and overland flow in previous studies based on the same methodology (Djodjic and Spännar 2012; Djodjic and Villa 2015; Djodjic et al. 2018) and in the present study. Djodjic et al. (2018) recognise that the presentation of the modelling results compared with farmers’ experience-based observations might be weak in statistical terms, and the same is true even for some results presented in this study (Fig. 5c). However, such maps are usually the most interesting results both for the farmers and for extension service workers, as an initial point for the discussion regarding proper placement of countermeasures. The experience so far is that such results must not be seen as a ground truth but are very useful to initiate discussions and focus mitigation efforts on relevant areas (Djodjic and Spännar 2012; Djodjic et al. 2018). We also achieved high geographical coverage of modelling results (> 90% of Swedish agricultural land) in the present study. Thus, the necessary preconditions for refinement and fine-tuning of existing countermeasures such as riparian buffer strips, P ponds and constructed wetlands are now available. Our modelled erosion risk maps could be used as an important starting point to discuss selection and placement of appropriate countermeasures by farmers, advisors and water authorities. This might help to establish a common view on problems and mitigation needs. Additionally, erosion was very low on a large parts of the modelled area (Fig. 5a). As illustrated in Fig. 7, fields with sandy soils and low LS factor are not susceptible for particle mobilisation. The abatement efforts on such fields should focus on P sources and the loss of DP rather than on soil conservation practices and transport pathways (Djodjic et al. 2018).

However, evaluation of the spatial agreement between modelled and observed results also revealed some possible failures of the model to properly describe actual field conditions. One type of systematic error was due to misrepresentation of input data, an example being incorrect flow accumulation, which created erosion lines where roads intercept water courses and divert the flow. Instead of continuing under the road through the culvert, water may be diverted to run along the road until it finds its way back to the original water course. This is a consequence of the lack of adjustments of DEM to road culverts. This type of error may be significant locally and should be considered when the results are evaluated at field scale, in case the diverted flow runs across the field. Another example is inaccuracy in the soil distribution map, as observed in a part of southern Sweden where the area of silt soils was overestimated. However, the soil textural class map used here to describe arable soil (Söderström and Piikki 2016) is based on the digital soil mapping framework (Minasny and McBratney 2016), and has high precision due to use of gamma radiation data (^232^Th and ^40^K, with strong correlation to soil clay content), in combination with high-resolution elevation data. Therefore, these kind of errors should be rather limited. Another type of possible error is inaccurate parameterisation, for instance of soil properties illustrated by *K* and *p* values of silty clay loam (Fig. 6a, b). Further evaluation, testing and calibration of the model with measured data will enable these errors to be reduced. Finally, there are conceptual issues with the kind of simple model used here. Transport of SS and P occurs both with overland flow and through the soil, especially via preferential flow through macropores (Djodjic 2001). Here, we modelled only the overland flow, while transport through the soil was not determined. While this is obviously conceptually wrong, there are several justifying circumstances for the approach. First, we modelled the worst case scenario, in which overland flow is the dominant transport pathway. Second, the modelled erosion lines run to a great extent straight over the main tile drains and, where present, surface water inlets in the drainage system (Djodjic and Villa 2015). This shows that the high-resolution elevation data are capable of capturing even small depressions created during the installation of tile drains. It also shows that the model accurately describes where overland flow occurs, since the installation of surface water inlets is driven by the need for efficient removal of overland flow from the fields at risk. Third, areas in the vicinity or just above the main drain tiles, which were precisely identified by the modelled erosion lines, might be more hydrologically active and lead to high SS and P losses due to the active macropores in the drain backfill (Øygarden et al. 1997). The disturbed backfill soil over a tile drain represents a good pathway for generating rapid P transport (Ulén et al. 2012). Therefore, it may be important to consider the modelled erosion lines when discussing mitigation options, even when overland flow or erosion is not visible in the field.

Besides the pronounced spatial variability, there is also great temporal variation in SS losses in Sweden (Alström and Bergman 1990). An inevitable question is how important the rare and often rather short episodes of intense transport of SS are for the total loads of SS and P. As we demonstrated in this study, months with loads exceeding the 90th percentile of SS were responsible for around one-third of the water flow, more than half the total SS loads and close to half the TP and UP loads (Table 4). This was true at both field and catchment scale, although the average values tended to be higher at field scale. From a mitigation point of view, this implies that any countermeasures implemented need to be effective during such intense episodes.

Sharpley et al. (2015) stress the need to target mitigation measures at vulnerable landscapes, but advise caution when applying risk tools developed at one scale (e.g. a field) to another scale (e.g. a catchment). We believe that the high-resolution distributed modelling reported here might resolve both these issues. The risk maps we produced seem to work equally well at field (e.g. observation fields) and at least small catchment scale (e.g. small agricultural catchments). However, the model results were tested and compared to measurements only at these scales. Further testing and validation of here presented model and results are necessary to assess its performance, especially quantitative assessment, for large catchments. At this scale, contribution from other sources such as bank erosion, large point sources and urban diffuse sources may be important. Therefore, catchment-specific calibrations should be performed if the objective is to use quantitative values in the assessment of mitigation programmes and activities at river basin scale. In addition, the modelled erosion lines indicate the connectivity in the landscape and can be helpful in understanding water and solute transport at scales beyond field and farm boundaries. Water authorities and agricultural agencies can thereby gain insights to promote collaboration between farmers at the landscape level, in order to find the best solutions for problems that may manifest in one field, but have their cause further upstream.

## Conclusions

The spatial variation in nutrient loads, especially P transport from land to water recipients, might be both a problem and an opportunity. Accurate identification of critical source areas is critical for implementing efficient and cost-effective countermeasures. In this study, we demonstrated that it is possible to model erosion risk maps with both high resolution (2 m × 2 m) and large geographical coverage (220 279 km^2^). The problem of erosion can thereby be addressed in individual fields and on more than 90% of Swedish arable land with uniform methodology. High spatial agreement between modelled results and independent field observations of overland flow and erosion made by farmers in the pilot catchment Vege å was a precondition before modelling was scaled up to the whole southern half of Sweden. We also demonstrated that the modelled worst case scenario produced reasonable quantitative results comparable to measured 90th percentile values of SS loads at both field and small catchment scale. Furthermore, we demonstrated and discussed the relevance of SS loads for P losses based on results of two monitoring programmes, at field and catchment scale. We firmly believe that the erosion risk maps we created can be an important base for discussions between different stakeholders (farmers, Swedish Board of Agriculture, water authorities and county boards) to define and prioritise mitigation efforts. In particular, they can be useful in accurate placement of measures aimed at reducing erosion and overland flow as transport pathways for SS and P, such as riparian buffer strips, P ponds, wetlands, structure liming etc. Since some of these measures have already been implemented, it might be as important to identify non-vulnerable areas where these measures are ineffective and costly as it is to apply them in high-risk locations.

There are several possibilities for improvement of the modelling methodology and in fact development of dynamic modelling with possibilities to enable comparison of modelling results with measured time series is already ongoing. This will permit consideration of seasonal changes in driving variables (climate, vegetation and crop cover distribution, and management practices). The model should be extended to account not only for SS but also direct P losses, including both UP and DP.
